# Presence of Breeding Birds Improves Body Condition for a Crocodilian Nest Protector

**DOI:** 10.1371/journal.pone.0149572

**Published:** 2016-03-02

**Authors:** Lucas A. Nell, Peter C. Frederick, Frank J. Mazzotti, Kent A. Vliet, Laura A. Brandt

**Affiliations:** 1 Department of Wildlife Ecology and Conservation, University of Florida, Gainesville, Florida, United States of America; 2 Department of Wildlife Ecology and Conservation, Fort Lauderdale Research and Education Center, University of Florida, Davie, Florida, United States of America; 3 Department of Biology, University of Florida, Gainesville, Florida, United States of America; 4 United States Fish and Wildlife Service, Davie, Florida, United States of America; University of South Carolina, UNITED STATES

## Abstract

Ecological associations where one species enhances habitat for another nearby species (facilitations) shape fundamental community dynamics and can promote niche expansion, thereby influencing how and where species persist and coexist. For the many breeding birds facing high nest-predation pressure, enemy-free space can be gained by nesting near more formidable animals for physical protection. While the benefits to protected species seem well documented, very few studies have explored whether and how protector species are affected by nest protection associations. Long-legged wading birds (Pelecaniformes and Ciconiiformes) actively choose nesting sites above resident American alligators (*Alligator mississippiensis*), apparently to take advantage of the protection from mammalian nest predators that alligator presence offers. Previous research has shown that wading bird nesting colonies could provide substantial food for alligators in the form of dropped chicks. We compared alligator body condition in similar habitat with and without wading bird nesting colonies present. Alligator morphometric body condition indices were significantly higher in colony than in non-colony locations, an effect that was statistically independent of a range of environmental variables. Since colonially nesting birds and crocodilians co-occur in many tropical and subtropical wetlands, our results highlight a potentially widespread keystone process between two ecologically important species-groups. These findings suggest the interaction is highly beneficial for both groups of actors, and illustrate how selective pressures may have acted to form and reinforce a strongly positive ecological interaction.

## Introduction

Facilitation is a positive ecological exchange in which one species enhances habitat for another nearby species (*sensu* [1]). Identifying and assessing the strength of facilitative interactions has enriched our understanding of species coexistence/persistence (e.g., [2–6]), and of the factors shaping populations and communities (e.g., [7–9]). The potential for ecological facilitation to expand niche boundaries also challenges the long-held notion of species interactions necessarily causing niche shrinkage [10–12].

Creation of enemy-free space is one common currency of facilitative exchange, and theory predicts that this form of facilitation will occur most frequently in communities where members experience high consumer pressure (e.g., predation, herbivory) [13,14]. For many bird species nest predation is the greatest threat to reproductive success [15–19], so breeding birds may nest near more-formidable animals for physical protection (e.g., [20–23]). Despite the wealth of literature on nest protection associations (reviewed in [24–26]), only six papers assess costs/benefits to the protective species (“protectors” hereafter; Table 1). This research bias in avian nest protection associations mirrors that in facilitations more generally, as most facilitation research focuses on fitness effects to the partner ostensibly receiving benefits (but see [27]). In both cases a largely unilateral approach limits our understanding of how these interactions evolve and persist [1,26].

**Table 1:** Published results regarding fitness effects on protectors in avian nest protection associations.

Protector		Protectee	Effect on protector		
Order	Species	Species	Direction	Form	Source
Charadriiformes	Whimbrel (*Numenius phaeopus*)	Bar-tailed godwit (*Limosa lapponica*)	None	N/A	[28]
	3 species (family: Laridae)	Sand-colored nighthawk (*Chordeiles rupestris*)	(−)	Nest defense (higher cost)	[29]
Passeriformes	Rufous-fronted thornbird (*Phacellodomus rufifrons*)	> 10 species (orders: Passeriformes, Galliformes)	(+) ^a^	Nest defense (higher efficacy)	[30]
			(−) ^a^	Aggression / nest predation	
Falconiformes	Merlin (*Falco columbarius*)	Fieldfare (*Turdus pilaris*)	(+)	Nest defense (higher efficacy)	[31]
	Lesser kestrel (*Falco naumanni*)	Jackdaw (*Corvus monedula*)	(+)	Nest defense (lower cost)	[32]
Hymenoptera	Polistine wasp (*Ropalidia cincta*)	Red-cheeked cordonbleu (*Uraeginthus bengalus*)	None	N/A	[22]

^a^ The author could not draw definitive conclusions for effects of individual species due to small sample sizes.

Nutritional benefits to protectors have not been explored [26]. This is despite (1) many protectors commonly consuming young and eggs of the protected species (“protectees” hereafter) [26] and (2) aggregations of breeding birds often increasing local primary and secondary productivity, which could provide nutritive benefits to protectors [33–35]. Further, many colonially nesting birds lay more eggs than they can raise, and adjust brood size to fit available food resources through several processes of brood reduction (reviewed in [36]). This often amounts to 1–2 chicks being ejected alive or dead from each nest, providing a potentially substantial source of food for protectors, especially in concentrated nesting associations [37]. Thus colonies of breeding birds offer multiple avenues through which they could nutritionally benefit protectors that do not necessitate exploitation by either partner.

In this study we report on benefits for American alligators (*Alligator mississippiensis*) that associate with nesting colonies of long-legged wading birds (orders Ciconiiformes and Pelecaniformes: herons, egrets, ibises, storks, and spoonbills; “wading birds” hereafter). In mixed-species wading bird nesting colonies in the southeastern United States, medium-sized, arboreal, semiaquatic mammals such as North American raccoons (*Procyon lotor*) and Virginia opossums (*Didelphis virginiana*) present the greatest nest predation threat, and these birds have no evolved defences against such nest predators [38,39]. Recent research suggests that wading birds actively choose nesting sites above alligators, and that in wetlands, there is a mutually exclusive distribution of alligators and mammalian predators [40]. Together with evidence that alligators readily consume mammals [41–44], there is reasonably strong evidence that alligators deter mammalian nest predators, thereby greatly increasing reproductive success for nesting wading birds.

Wading bird nesting aggregations can substantially increase nearby nutrient deposition [45–47] and may enhance primary and secondary productivity as a result. Moreover, the quantity of food potentially available to scavengers from wading bird colonies via dead chicks is substantial, enough to theoretically support large populations of alligators [37]. Given the potential for significant energetic benefits to alligators in wading bird nesting colonies, we predicted that alligators associated with wading bird colonies will have higher body condition indices than alligators in similar habitat without colonies.

## Materials and Methods

### Ethics Statement

All animal use was approved by the University of Florida’s Institute of Food and Agricultural Sciences Animal Research Committee under Approval No. 007-13WEC. All field work and sample collection was performed under Florida Fish and Wildlife Conservation Commission Scientific Collecting Permit No. SPGS-13-58 and United States Fish and Wildlife Service Arthur R. Marshall Loxahatchee National Wildlife Refuge Special Use Permit No. B14-006. All efforts were made to minimize stress to animals during measurements and tissue sampling, and study animals were released at the point of capture immediately after processing (within ~1 hour).

### Study Sites

**Fig 1:**
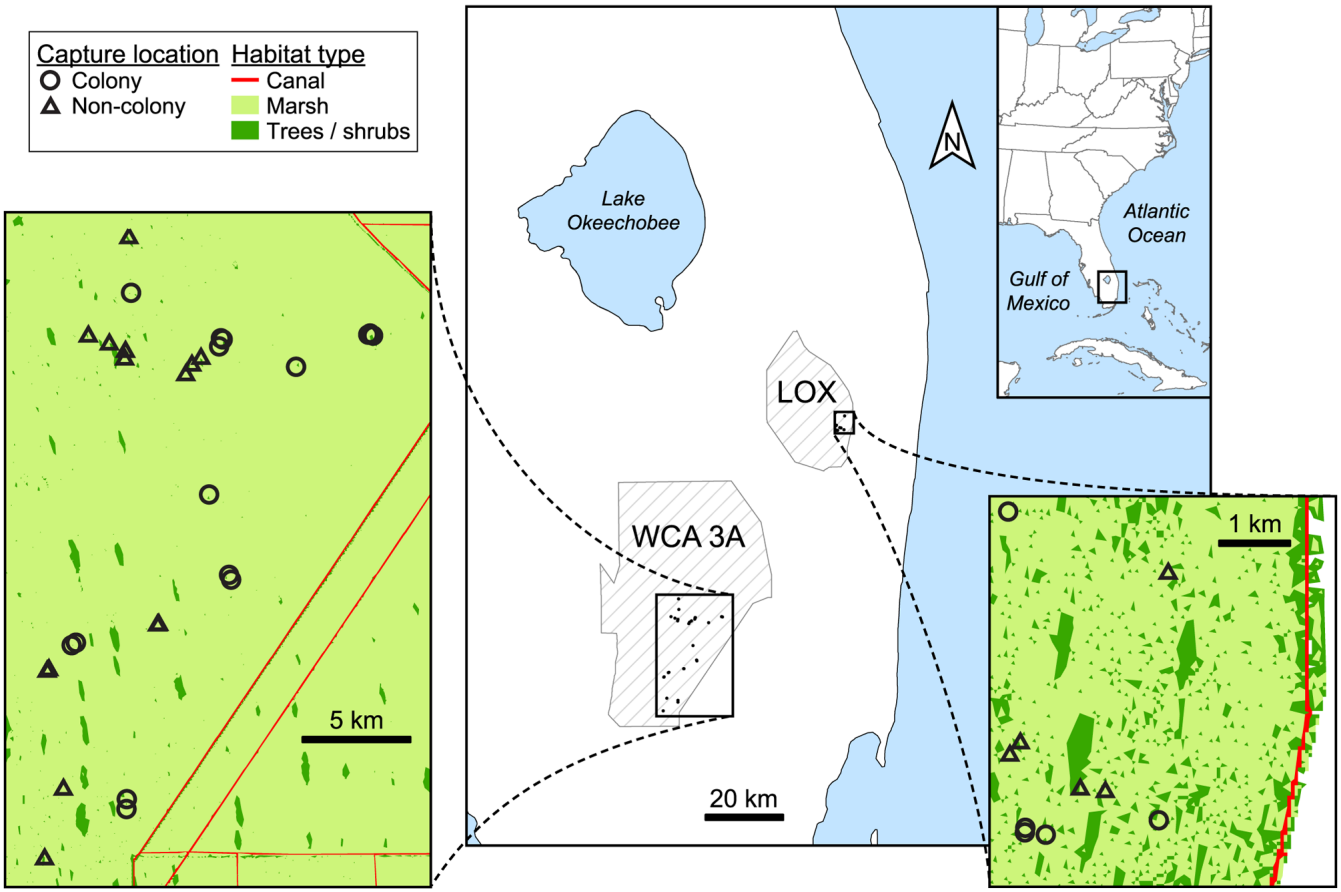
Map of the study area. Locations of Water Conservation Area 3A (WCA 3A) and Arthur R. Marshall Loxahatchee National Wildlife Refuge (LOX) in the Everglades of Florida, USA, and inset maps of adult female alligator capture sites. In capture-site maps, habitat type shows the densities of tree islands (clusters of trees / shrubs) and locations of canals near capture sites; white areas in the LOX inset are non-habitat. Open-source data for habitat type were obtained from the Ecological Modeling Team at Everglades National Park: http://simglades.org/.

This study took place in the Everglades of Florida, USA: Water Conservation Area 3A (WCA 3A; 25.961°, −80.701°) in Miami-Dade and Broward Counties, and Arthur R. Marshall Loxahatchee National Wildlife Refuge (LOX; 26.489°, −80.337°) in Palm Beach County (Fig 1). These freshwater marshes are a mosaic of habitats including deeper-water sloughs, wet prairies, sawgrass (*Cladium jamaicense*) strands, and elevated tree islands. Hydrologic conditions fluctuate seasonally (by ~40 to >100 cm, depending on the area and year) with lowest water depths during the November to May dry season. Wading bird nesting colonies are predominantly located in inundated, lower-elevation islands with the longest hydroperiods [39]. In WCA 3A these islands are typically dominated by coastalplain willows (*Salix caroliniana*), while in LOX most are comprised of swamp bay (*Persea palustris*), dahoon holly (*Ilex cassine*), and other trees/shrubs.

Alligators were captured in sloughs surrounding tree islands that were designated as either colony or non-colony sites as follows. We used data from systematic, 100% coverage aerial and ground surveys conducted during the nesting season to locate active wading bird nesting colonies (see [48] for details). Approximately half of the alligators were caught within 200 m of islands with 20–800 nests of wading birds (colony sites); the other half were caught near islands that (1) were > 1 km from the nearest active colony and (2) had not been occupied by nesting birds in the previous 5 years (non-colony sites). Both colony and non-colony sites were > 1 km from the nearest canal because these unnatural areas affect alligator size-distributions [49] and fish abundances [50]. The distances of 1 km and 200 m were derived from radio telemetry studies that estimated daily linear movements and home range sizes for alligators in our study area [51]. For non-colony sites in WCA 3A, we used Google Earth [52] to identify tree islands bordered by the same canals, of similar size and vegetation composition, and within 5–10 km of each colony site. In LOX, the ubiquity of tree islands and their relatively similar size allowed us to use any location > 1 km away from the nearest nesting colony as a non-colony site.

### Alligator Sampling

We captured adult and subadult (≥ 125 cm total length) alligators by noose or hand from an airboat between 2000 and 0530 hours in June of 2013 and 2014. We began captures immediately after bird nesting had largely been completed, to minimize our disturbance to active nests. Because female alligators have smaller home ranges and move less than males in the Everglades [51], we assumed that female body condition should be more reflective of food opportunities from an individual tree island than male body condition. Because of this and the fact that adult females have the greatest influence upon alligator population dynamics [53], we only used females for analyses. We determined sex by cloacal examination and recorded geographical coordinates of capture location. We measured snout-vent length dorsally (± 0.1 cm) and body mass (± 0.5 kg) using a spring scale.

For hematological indices, we extracted 5 mL of blood from the cranial sinus of each captured alligator using a 20-G needle, which was immediately transferred to either sodium (2013) or lithium (2014) heparin Vacutainer tubes and centrifuged (3,400 rpm for 10 minutes). Because this location of blood draw is subject to dilution from lymph or cerebrospinal fluid, we cross-referenced plasma total protein concentrations from other alligator studies [54]. Blood samples were all taken within fifteen minutes of initial noosing or hand-capture. We pipetted the plasma into vials and stored them in a cooler of ice until we returned from the field. The plasma was stored in a freezer at −20°C (2013) [55] or on dry ice or in a −80°C freezer (2014) until the tests were run. The frozen plasma was transported to the Avian & Wildlife Laboratory, University of Miami (Miami, FL) for analyses.

### Condition Indices

Four hematological markers were used as indicators of nutritional status, which are collectively called intermediary plasma metabolites (IPMs). The IPMs glucose, triglycerides, β-hydroxy-butyrate (BHB), and uric acid have been used in birds and crocodilians to detect nutritional deficiencies [55–58]. In these studies, elevated plasma concentrations of glucose and triglycerides indicated little to no starvation, while elevated BHB and drops in glucose and triglycerides signified intermediate starvation; increased uric acid was associated with severe starvation. We predicted alligators in nesting colonies would have higher plasma glucose and triglycerides, and lower BHB and uric acid than those not found in colonies.

Morphometric body condition indices can be used to indicate an animal’s energy reserves relative to size [59]. These indices have been shown to be positively correlated with reproductive success in birds [60], turtles [61], and snakes [62]. They have also been used as reliable, efficient indictors of alligator population health [63–66]. Fulton’s condition factor (*K*) was used because it has been used previously in alligators; *K* was calculated as such: *K* = *M* * *SVL*^−3^ * 10^5^, where *M* is mass and *SVL* is snout-vent length [67–69].

Because *K* may bias condition scores due to allometry [70,71], the scaled mass index (${\hat{M}}_{i}$) was also used. ${\hat{M}}_{i}$ was calculated using the following equation: ${\hat{M}}_{i}$ = *M*_*i*_ * [*SVL*_*0*_ / *SVL*_*i*_] ^*b*_*SMA*_, where *M*_*i*_ and *SVL*_*i*_ are mass and snout-vent length of individual *i* respectively, *SVL*_*0*_ is an arbitrary length (we used 100 cm), and *b*_*SMA*_ is the scaling exponent as determined by a standardized major axis (SMA) regression of ln(*M*) on ln(*SVL*) [71,72]. For the SMA regression we used a reference population of female alligators caught in our study area from 1999–2014 (*n* = 565, mean ± SD [range], *M*: 16.38 kg ± 11.10 [0.82–56.00], *SVL*: 85.55 cm ± 21.39 [35.0–135.9]). We could not determine whether individuals from this reference population were associated with nesting colonies. However, since Everglades alligators are particularly thin [64,73], a study-area-specific reference population better-informed our prediction of how mass scales with length in this population. We also checked that growth was approximately isometric (i.e., mass ∝ length^3^) within our capture sample by conducting an ordinary least squares (OLS) regression of ln(*M*) on ln(*SVL*) and testing the hypothesis that the regression coefficient for length was significantly different from three [74].

### Environmental Covariates

We included four environmental covariates in our models: (1) yearly minimum water depth, (2) range in water depth, (3) tree island area, and (4) counts of nearby alligator-maintained ponds (alligator holes). (1) We predicted a unimodal relationship between alligator body condition and yearly minimum water depth [49,50,69]. In the Everglades, low water levels confine prey and make them more available for capture by alligators, yet in particularly dry years alligator populations decline [75]. (2) Greater range in water depth has been shown to increase wetland productivity [76–79], (3) tree islands are nutrient hotspots in the Everglades that increase local productivity [80–83], and (4) during low water conditions in the Everglades, fish and other aquatic prey congregate into alligator holes [84,85]; we predict each of factors 2–4 to have positive effects on alligator body condition through higher local prey abundance. From this information, we created a suite of *a priori* hypotheses upon which we based our statistical models (S1 Table).

From the Everglades Depth Estimation Network project website (http://sofia.usgs.gov/eden/) we extracted predicted water depths for the 400×400 m grid cells in which each capture occurred; this water depth model has been validated to RMSE = 3.3 cm [86]. We used water depths from within each capture’s calendar year for minimum yearly water depth, and from ≤ 365 days prior to capture for range in water depth. From the Ecological Modeling Team at Everglades National Park (http://simglades.org/) we downloaded habitat type by 50×50 m grid cells (for tree island area calculations) and locations of alligator holes in our study area (see [49] for how these data were derived). For tree island area, we calculated the proportion of nearby grid cells that we categorized as tree island, while for alligator holes we took counts of nearby holes. We only considered habitat-type cells or alligator holes within sqrt{400^2^ / π} m of each capture location, so that the area concerned was the same as for the water-depth data.

### Statistical Analyses

All analyses were conducted in R 3.1.2 (R Core Team 2014). Due to non-normality of response variables, we used one-way percentile bootstrap hypothesis tests (10^6^ simulations) [87,88] of H_0_: μ_*nc*_ ≥ μ_*c*_, where *c* is colony and *nc* is non-colony, for *K*, ${\hat{M}}_{i}$, and glucose, and H_0_: μ_*nc*_ ≤ μ_*c*_ for uric acid. Influential points were determined using plots of jackknife influence values. Triglycerides and BHB had samples with undetected levels, so we used the *G*^*ρ*^ family [89] equivalent to the Peto and Peto [90] modification of the Gehan-Wilcoxon test, to test for differences in the empirical cumulative distribution functions for colony and non-colony values [91]. To conduct these tests, we used the R package ‘*NADA*’ [92], which adjusts routines from the package ‘*survival*’ [93] to handle left-censored data.

If a variable was different between colony and non-colony females, we assessed potential covariate effects using linear models. Diagnostic plots indicated heteroscedasticity and non-normality in some models, so we used Box-Cox [94] data transformations. Plots also indicated influential points, so we employed robust regression techniques: Huber’s [95] M-estimator and iterated median absolute deviations. These techniques were carried out by the functions ‘*boxcox*’ and ‘*rlm*’ in the ‘*MASS*’ package [96]. We evaluated support for robust linear models (RLMs) using the second-order variant of Akaike’s Information Criterion (*AIC*_*c*_), the difference in *AIC*_*c*_ between model *i* and the top model (Δ_*i*_), and Akaike weights (*w*_*i*_); the latter represents Pr(model_*i*_ is the best model | data) [97].

## Results

We captured thirty-nine female alligators (20 colony, 19 non-colony), ranging from 7.5 to 46.0 kg (mean ± SD, 21.3 kg ± 9.7) total mass and 146.6 to 239.1 cm (194.1 cm ± 27.1) total length. Because female alligators in the Everglades can reproduce at 1.5 m total length [64], we considered all individuals potentially reproductively active and hereafter refer to them as adults.

The bird nesting colonies at which we captured alligators were primarily comprised of great egrets (*Ardea alba*), with smaller numbers of little blue herons (*Egretta caerulea*), tricolored herons (*E*. *tricolor*), snowy egrets (*E*. *thula*), and anhingas (*Anhinga anhinga*). Yearly minimum water depth was much deeper for colony (25.5 cm ± 15.8) than non-colony (14.9 cm ± 12.5) sites in WCA 3A (one-way Welch’s *t*-test: *t*_*26*.*4*_ = 2.02, *P* = 0.027), while it was similar in LOX (colony: 13.2 cm ± 8.26, non-colony: 11.4 cm ± 7.40, *t*_*7*.*91*_ = 0.363, *P* = 0.363).

**Fig 2:**
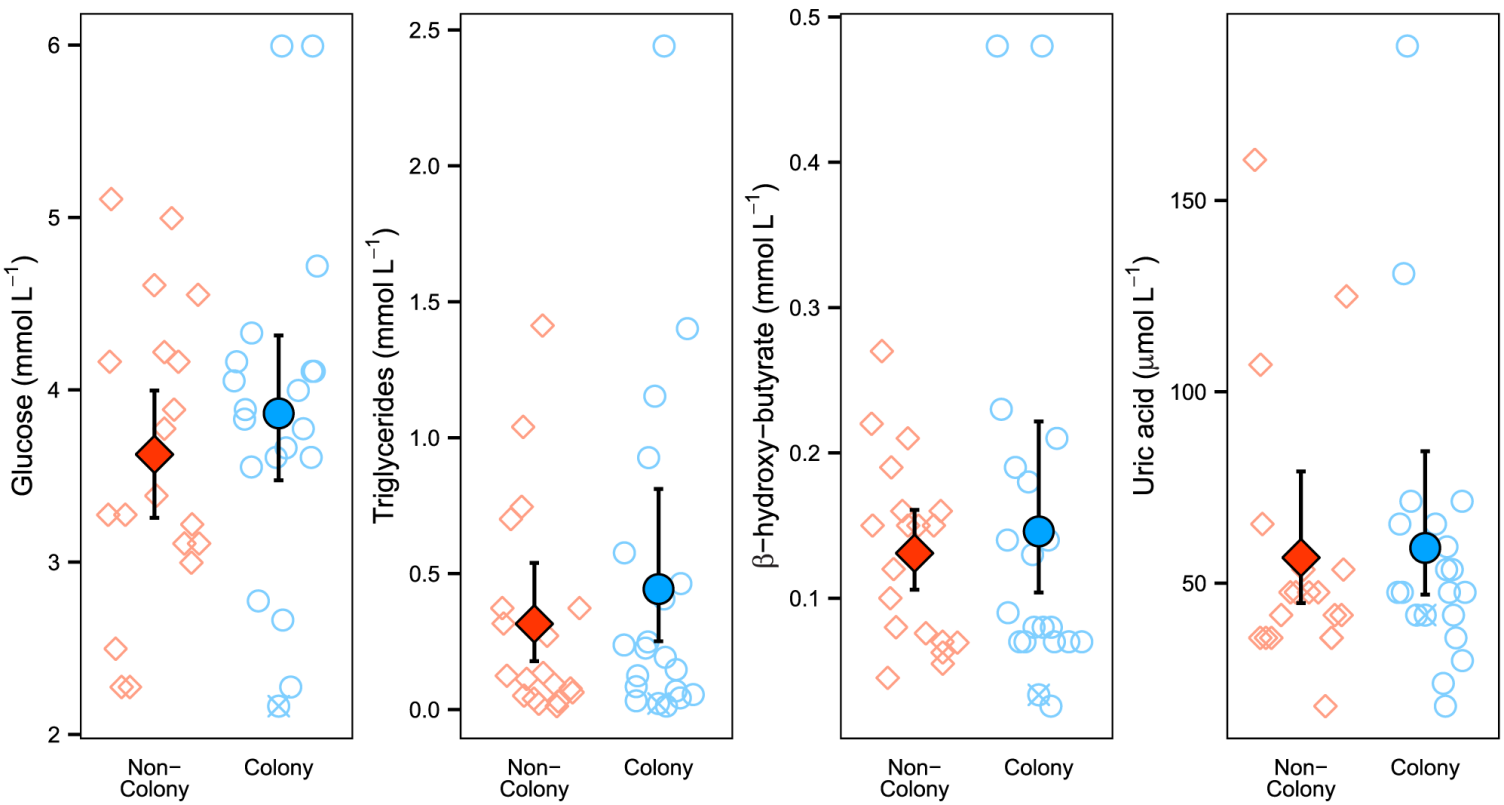
Intermediary plasma metabolites (IPMs) for colony and non-colony alligators. Comparison of the IPMs glucose, triglycerides, β-hydroxy-butyrate (BHB), and uric acid for adult female alligators caught near Everglades tree islands with and without wading bird nesting colonies present. Error bars are CI_95%_ via bias-corrected and accelerated bootstrapping; crossed points indicate the individual caught with a broken tail. For triglycerides and BHB, censored values were replaced with model estimates from separate regressions on order statistics for colony and non-colony subpopulations.

Glucose (*P* = 0.207) and uric acid (*P* = 0.585) were not significantly different between colony and non-colony females, and hypothesis tests for left-censored data showed no differences in triglycerides (χ^2^_1_ = 0.647, *P* = 0.421) or BHB (χ^2^_1_ = 7.95×10^−4^, *P* = 0.978) (Fig 2). Total protein values (43.9 ± 12.7 g L^−1^) were similar to those from other alligator studies [54,98], which suggests that our samples were not markedly diluted with lymph or cerebrospinal fluid [54].

**Fig 3:**
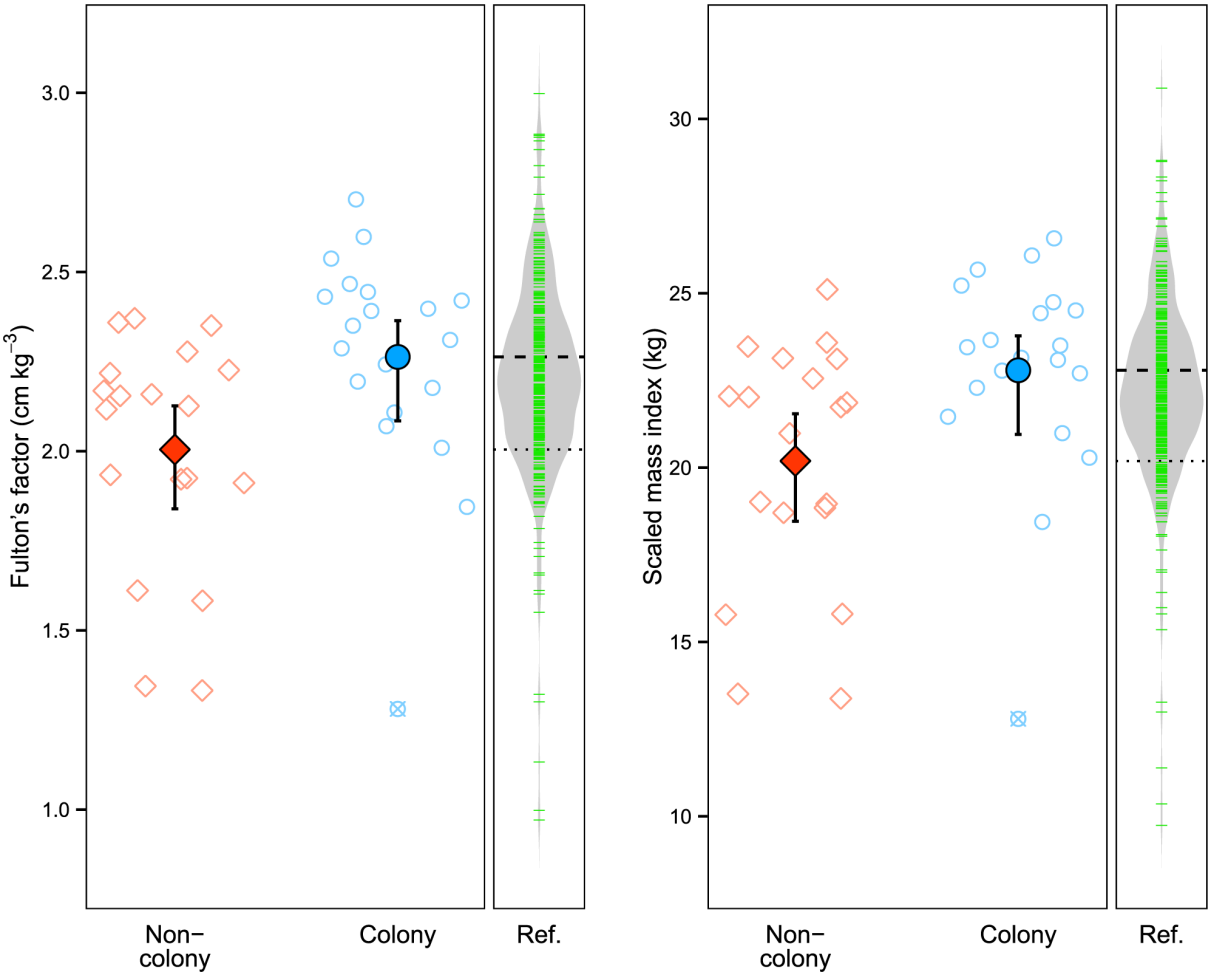
Morphometric body condition for colony and non-colony alligators. Comparison of morphometric body condition (Fulton’s factor, *K*, and scaled mass index, ${\hat{M}}_{i}$) for adult female alligators caught near Everglades tree islands with and without wading bird nesting colonies present. Error bars are CI_95%_ via bias-corrected and accelerated bootstrapping, and crossed points indicate the individual caught with a broken tail. Beanplots represent values from the reference population of alligators caught in our study area between 1999–2014, filtered for those within the range of snout-vent lengths from our captured females (*n* = 387); dashed and dotted lines are located at the mean values for colony and non-colony alligators, respectively.

Both morphometric indices were significantly higher (*K*: *P* = 0.008, ${\hat{M}}_{i}$: *P* = 0.010) in colony (*K* = 2.26 cm kg^−3^ ± 0.310, ${\hat{M}}_{i}$ = 22.79 kg ± 3.09) than non-colony (*K* = 2.00 cm kg^−3^ ± 0.325, ${\hat{M}}_{i}$ = 20.19 kg ± 3.48) female alligators (Fig 3). Jackknife-after-bootstrap plots indicated one colony female as a potential outlier for these bootstrap hypothesis tests, an individual with a broken tail that likely influenced its ability to forage. As we had biological and statistical evidence that this point was a likely outlier, we identified it in Figs 2 and 3.

The 5 RLMs for *K* that included colony presence as a predictor represented the 5 most highly supported models (Table 2). The highest-supported model also included range in water depth and alligator hole counts as additive terms (*w*_*i*_ = 0.73), while the second-best model included colony presence as the lone predictor (*w*_*i*_ = 0.13).

**Table 2:** Model selection on RLMs predicting alligator body condition (Fulton’s factor, *K*).

Model	*AIC*_*c*_	Δ_*i*_	*w*_*i*_	*k*
Colony presence + Water depth range + Alligator holes	215.80	0.00	0.73	5
Colony presence	219.23	3.43	0.13	3
Colony presence + Water depth range	220.38	4.58	0.07	4
Colony presence + Tree island area	221.83	6.02	0.04	4
Colony presence × Minimum water depth^†^	224.49	8.69	0.01	7
Water depth range	225.13	9.33	0.01	3
Tree island area	226.91	11.10	0.00	3
Minimum water depth^†^ × Water depth range	227.05	11.25	0.00	7
Minimum water depth^†^	229.19	13.38	0.00	4
Minimum water depth^†^ × Tree island area	231.73	15.93	0.00	7
Minimum water depth^†^ × Alligator holes	236.19	20.39	0.00	7

*AIC*_*c*_, second-order variant of Akaike’s Information Criterion; Δ_*i*_, difference in *AIC*_*c*_ between model *i* and the top model; *w*_*i*_, relative likelihood of model *i* [i.e., Pr(model_*i*_ is the best model | data)]; *k*, number of model parameters

^†^ Quadratic term included

**Fig 4:**
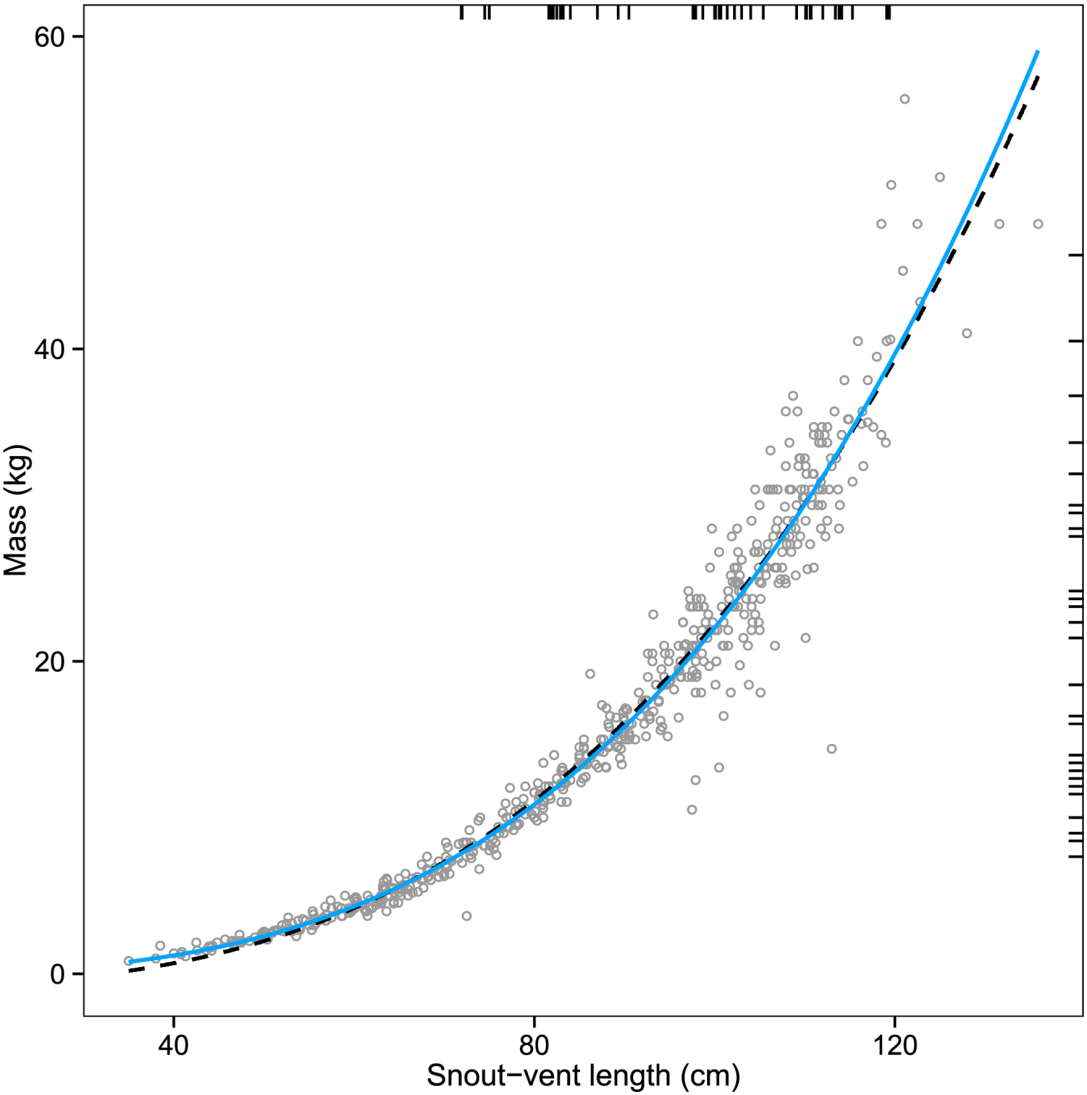
Mass versus length for the reference population. Plot of mass against snout-vent length for a reference population of alligators caught in the study area from 1999–2014 (*n* = 565). Regression lines are for standardized major axis (dashed) and ordinary least squares (solid) regressions, and rug plots along vertical and horizontal axes represent masses and snout-vent lengths respectively for alligators in this study.

${\hat{M}}_{i}$ showed similar results as *K* with respect to model selection (S2 Table). The SMA regression of ln(*M*) on ln(*SVL*) using the reference population (*n* = 565, *r*^2^ = 0.981, *P* << 0.001) indicated a scaling exponent of 3.20 (CI_95%_ = 3.17–3.24) for the calculation of ${\hat{M}}_{i}$. However, the *M*–*SVL* relationship within the population of animals captured was not significantly different from that predicted for isometric growth (OLS regression: *t*_37_ = −0.471, *P* = 0.354, coefficient CI_95%_ = 2.51–3.30), nor did the curves for *M* ∝ *SVL*^3^ differ markedly from *M* ∝ *SVL*^3.20^ within the size-range of alligators captured herein (Fig 4).

## Discussion

Our results using morphometric indices are consistent with the hypothesis that wading birds are facilitators of alligators by providing localized nutritional subsidies. Both Fulton’s factor (*K*) and the scaled mass index (${\hat{M}}_{i}$) indicated that alligators near wading bird nesting colonies were in better body condition than those in similar habitat without active colonies (Fig 3), an effect that was statistically independent of environmental factors (Table 2 and S2 Table). Because (i) the results were similar for *K* and ${\hat{M}}_{i}$, (ii) the mass—length relationship within our capture population did not differ significantly from 3 (CI_95%_ = 2.51–3.30; Fig 4), and (iii) *K* afforded us opportunities to compare to past studies, we hereafter only use *K* as our morphometric condition index and refer to it as simply “body condition.” However, we recommend future studies to utilize the scaled mass index if analyzing a wide size-range of alligators.

Results from IPMs (glucose, triglycerides, BHB, and uric acid) did not support our hypothesis (Fig 2), which contrasts previous work using IPMs in Yacare caiman (*Caiman crocodilus yacare*) [55]. However, their lower-condition population was caiman on dry land, where they likely have few or no feeding opportunities. All alligators in our study were captured in aquatic habitat and were not likely to have been completely deprived of food. The effects of intermittent feeding on IPMs is unclear in crocodilians, and even our most emaciated alligator via morphometric indices had inconclusive IPM results (crossed points in Figs 2 and 3). Additional studies in birds and squamates with consistent IPM results are typically either on laboratory-starved animals (e.g., [57]) or wild populations suffering extreme resource limitations and/or environmental contamination (e.g., [56,58]). Combined with the differences we found using morphometric indices, we infer that these blood parameters are only sensitive enough to discern severe nutritional differences.

Indeed, morphometric body condition results suggest biologically relevant effects of nesting colonies. Using those female alligators from our reference population that were within the range of snout-vent lengths reported herein (*n* = 387), mean body condition for colony-associated females we captured ranked as the 63^rd^ percentile, while that for non-colony females ranked as the 17^th^ (Fig 3). Moreover, the observed disparity between colony and non-colony alligator body condition (13%) is greater than the differences in pre-breeding-season condition for blue petrel (*Halobaena caerulea*) females that did and did not “decide” to breed (11%) [60], and in green turtle (*Chelonia mydas*) condition for those caught in years least and most associated with density-dependent reductions in growth rates (~8%) [99]. Thus the body condition difference we report is likely large enough to be associated with breeding potential for these subpopulations of alligators.

In seasonal wetlands in the Everglades, crocodilian food availability is thought to increase during dry months [69,73,100]. The magnitude of the increase in alligator body condition through colony association was similar to body condition changes effected through dry versus wet seasons (13%) [69] and two years of elevated water levels (−15%) [101]. Yet the differences we reported occurred despite colony sites having equal or greater water depths than non-colony sites, which might suggest that, on the scale of an individual tree island, colony association can buffer the effects of hydrology on nearby alligators. Alternatively, size and dry-season fish abundance are positively correlated for aquatic refuge (i.e., drought-resistant) sites in the Everglades [50], so the deeper modeled water depths near colonies in WCA 3A might indicate larger, deeper-water refuge sites with greater prey abundance. However, the colony-associated difference in body condition was greater for captures in LOX (16.7%), where water depths were similar between colony and non-colony sites, than in WCA 3A (11.6%). This sheds doubt upon deeper refuge sites near colonies causing the observed differences.

The apparent mutually facilitative association between nesting wading birds and alligators is a novel nest protection association, as the protector receives potentially substantial nutritional benefits from the protectee. The magnitude of benefits demonstrated here indicates that for alligators there should be selective pressure toward behaviors that enhance the benefits they derive from this association. We hypothesize that alligators are attracted to and, given their territorial behavior, may even compete for territories that include wading bird colonies. We predict from this that alligators should display movements towards bird colonies upon their formation, and alligators occupying colonies should be larger and/or occur more densely than in non-colony sites. Testing the above predictions will be key to understanding how the close spatial association between these two species arises, specifically whether alligator behavior has interactive effects with wading birds’ previously demonstrated attraction to alligator-present sites [40].

In comparison to other nest protectors described (Table 1; Appendix 1 in [26]) alligators are much larger-bodied, more indiscriminate in food choice, and less capable of reaching nests in trees. This has two important effects which could serve to reinforce this relationship: First, a comparatively large portion of nutrition from breeding birds should directly or indirectly reach alligators, as the latter could take advantage of an increase in small aquatic prey that may be fuelled by bird-guano deposition yet are large enough to consume chicks of all sizes. Brood reduction is common in all wading birds [36,102,103], and the resulting chick carcasses from wading bird nesting colonies represent the most substantive food source for associate alligators [37]. Because alligators can utilize this food source, brood reduction by wading birds is likely another vital component of this relationship, providing a steady flow of nutrients from protectee to protector.

Second, the risk of alligator predation on wading birds should drop quickly to zero with distance of nests above alligators. Although crocodilians are capable of jumping vertically, even large adult alligators are unlikely to reach heights of over ~1.5–2 m [104,105], particularly in the relatively shallow water and thick vegetation within tree islands. Nesting at such a height is a relatively small price to pay if the remuneration for birds is protection from nest predation by mammalian predators, and the ability of birds to nest directly over alligators with relatively little threat of predation should allow for a close connection between protector and protectee. This would increase the likelihood of both partners receiving benefits, as alligators in close proximity to bird nests should be more likely to (1) detect and consume fallen chick carcasses and (2) deter mammalian predators.

The oligotrophic Everglades is a particularly harsh environment for crocodilians, as it induces high energetic demands for resident ectotherms but offers a relatively poor food base [64,73,106]. We suggest that further research should seek to replicate these findings in other wetlands less energetically demanding for crocodilians. We also suggest that the basic mechanisms of this apparent two-way ecological facilitation could apply broadly to analogous species-groups of colonially nesting wetland birds and crocodilians in many other tropical and subtropical regions (e.g., floodplains and wetlands of southeastern USA, Western Australia, India, Africa, the Amazon, the Pantanal, and the Llanos).

## Supporting Information

S1 Fig Images of alligators positioned under wading bird chicks. Alligators (red) are observed under white ibis chicks (blue) in wading bird nesting colonies (A) “Alley North” (26.201°, −80.529°) and (B) “163” (25.773°, −80.833°). (C) In the image from colony “Tamiami West” (25.758°, −80.545°), the camera is facing down from an anhinga nest. Reprinted under a CC BY license, with permission from (A) Nicholas E. Vitale and (B, C) Lucas A. Nell, original copyrights 2014.
(PDF)

S1 Table Our *a priori* hypotheses on factors influencing alligator body condition. (PDF)

S2 TableModel selection on RLMs predicting alligator standardized mass index (${\hat{M}}_{i}$).*AIC*_*c*_, second-order variant of Akaike’s Information Criterion; Δ_*i*_, difference in *AIC*_*c*_ between model *i* and the top model; *w*_*i*_, relative likelihood of model *i* [i.e., Pr(model_*i*_ is the best model | data)]; *k*, number of model parameters.(PDF)

S3 Table Summary of data from alligators captured near colony and non-colony tree islands. (XLSX)

S4 Table Morphological data from a reference population of alligators. (XLSX)
