# Supplementary figures and images for: Presence of Breeding Birds Improves Body Condition for a Crocodilian Nest Protector
Source: PLoS One. 2016 Mar 2;11(3):e0149572. doi: 10.1371/journal.pone.0149572 (PMC4775066; doi:10.1371/journal.pone.0149572)

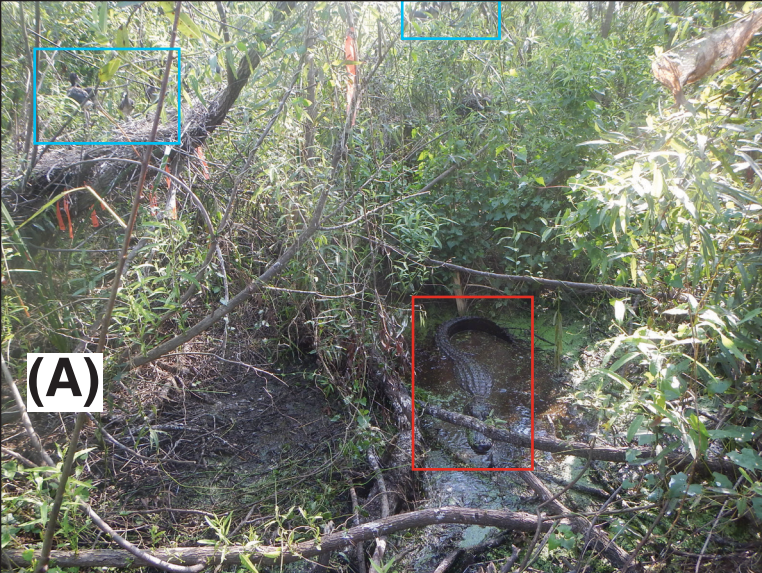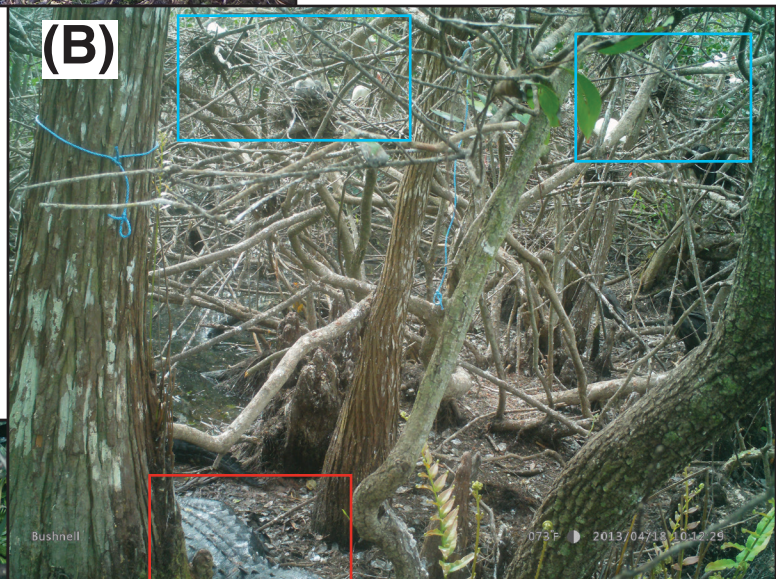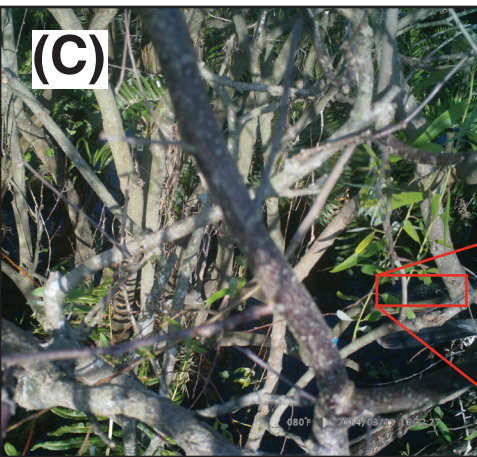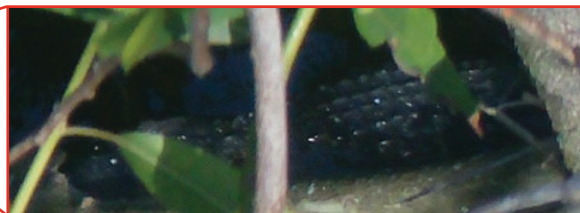

Supplement: S1 Fig — Alligators (red) are observed under white ibis chicks (blue) in wading bird nesting colonies (A) “Alley North” (26.201°, −80.529°) and (B) “163” (25.773°, −80.833°). (C) In the image from colony “Tamiami West” (25.758°, −80.545°), the camera is facing down from an anhinga nest. Reprinted under a CC BY license, with permission from (A) Nicholas E. Vitale and (B, C) Lucas A. Nell, original copyrights 2014. (PDF) [file pone.0149572.s001.pdf]
