# Supplementary material for: Presence of Breeding Birds Improves Body Condition for a Crocodilian Nest Protector
Source: PLoS One. 2016 Mar 2;11(3):e0149572. doi: 10.1371/journal.pone.0149572 (PMC4775066; doi:10.1371/journal.pone.0149572)
Supplement: S1 Table — (PDF) [file pone.0149572.s002.pdf]

Terms†	Description	Refs.
Colony presence	Wading bird nesting colony presence may increase alligator body condition due to the large quantities of supplemental food they provide.	[1]
Minimum water depth†	We predict alligator body condition to have a unimodal response to minimum water depth: Moderately low water depths (<40 cm) increase food availability via spatial confinement of prey. Yet low water depths (<15 cm) limit alligators' ability to move and forage, and in particularly dry years/areas (≤0 cm), alligator aquatic refuge becomes limited; both of these especially dry conditions presumably increase stress and decrease body condition.	[2–5]
Water depth range	Greater range in water depth can increase wetland productivity, which could increase prey abundance and improve alligator body condition.	[6–9]
Tree island area	Nutrient-dense tree islands increase local productivity, so alligators in habitat with greater amounts of tree island area may have greater food opportunities.	[10–13]
Minimum water depth† × Water depth range	We include an interactive term between minimum water depth and range in water depth because in deep water where food availability is low, we predict high productivity driven by range in water depth to have a pronounced effect.	[2–9]
Minimum water depth† × Tree island area	We predict an interaction between minimum water depth and tree island area, as potential increases in productivity associated with tree island area should have a reduced effect during drier conditions when alligators have lower mobility and cannot take advantage of increases in resources.	[2–5, 10–13]
Minimum water depth† × Alligator holes	In low water conditions, aquatic prey congregates in alligator holes, providing ample food for alligators and presumably improving body condition. We predict an interaction with minimum water depth, as alligator holes would probably have a more pronounced effect in drier conditions.	[2–5, 14, 15]
Colony presence × Minimum water depth†	We predict colony presence to interact with minimum water depth, as food from colonies probably has a pronounced effect when food availability is otherwise low (in deeper-water areas). Moreover, dry conditions (<15 cm) may reduce colonies' effects, as they would limit alligator movement.	[1–5]
Colony presence + Water depth range	Both colony presence and productivity via range in water depth may have additive effects on alligator body condition due to increased food opportunities.	[1, 6–9]
Colony presence + Water depth range + Alligator holes	Here we predict that alligators would have highest body condition in habitat containing nesting colonies, large numbers of alligator holes, and great range in water depth.	[1, 6–9, 14, 15]
Colony presence + Tree island area	Greater prey abundance due to nutrients from tree islands might combine with food from colonies to provide ample food for alligators, thereby improving their body condition.	[1, 10–13]

† Quadratic term included

References

1. Nell LA, Frederick PC. Fallen nestlings and regurgitant as mechanisms of nutrient transfer from nesting wading birds to crocodilians. Wetlands. 2015;35(4):723–732.

2. Fujisaki I, Rice KG, Pearlstine LG, Mazzotti FJ. Relationship between body condition of American alligators and water depth in the Everglades, Florida. Hydrobiologia. 2009;635(1):329–338.

3. Parkos III JJ, Ruetz III CR, Trexler JC. Disturbance regime and limits on benefits of refuge use for fishes in a fluctuating hydroscape. Oikos. 2011;120(10):1519–1530.

4. Shinde D, Pearlstine LG, Brandt LA, Mazzotti FJ, Parry MW, Jeffery BM, et al. Alligator production suitability index model (GATOR– PSIM v. 2.0): ecological and design documentation. Homestead, FL, USA; 2014.

5. Waddle JH, Brandt LA, Jeffery BM, Mazzotti FJ. Dry years decrease abundance of American alligators in the Florida Everglades. Wetlands. 2015;35(5):865–875.

6. Bedford B, Labisky R, van der Valk A, Volin J. Ecological effects of extreme hydrological events on the Greater Everglades; 2012.

7. Kushlan JA. Avian use of fluctuating wetlands. In: Sharitz RR, Gibbons JW, editors. Freshwater wetlands and wildlife. Oak Ridge, TN: US Department of Energy; 1989. p. 593–604.

8. Leira M, Cantonati M. Effects of water-level fluctuations on lakes: an annotated bibliography. Hydrobiologia. 2008;613(1):171–184.

9. Nuttle WK. Measurement of wetland hydroperiod using harmonic analysis. Wetlands. 1997;17(1):82–89.

10. Gaiser EE, Trexler JC, Wetzel PR. The Florida Everglades. In: Batzer DP, Baldwin AH, editors. Wetland habitats of North America: ecology and conservation concerns. Berkeley, CA: University of California Press; 2012. p. 231–252.

11. Givnish TJ, Volin JC, Owen VD, Volin VC, Muss JD, Glaser PH. Vegetation differentiation in the patterned landscape of the central Everglades: importance of local and landscape drivers. Global Ecology and Biogeography. 2008;17(3):384–402.

12. Ross MS, Sah JP. Forest resource islands in a sub-tropical marsh: soil–site relationships in Everglades hardwood hammocks. Ecosystems. 2011;14(4):632–645.

13. Wetzel PR, van der Valk AG, Newman S, Gawlik DE, Troxler TG, Coronado-Molina CA, et al. Maintaining tree islands in the Florida Everglades: nutrient redistribution is the key. Frontiers in Ecology and the Environment. 2005;3(7):370–376.

14. Kushlan JA. Observations on the role of the American alligator (*Alligator mississippiensis*) in the southern Florida wetlands. Copeia. 1974;1974(4):993–996.

15. Palmer ML, Mazzotti FJ. Structure of Everglades alligator holes. Wetlands. 2004;24(1):115–122.
