# Supplementary material for: Presence of Breeding Birds Improves Body Condition for a Crocodilian Nest Protector
Source: PLoS One. 2016 Mar 2;11(3):e0149572. doi: 10.1371/journal.pone.0149572 (PMC4775066; doi:10.1371/journal.pone.0149572)
Supplement: S2 Table — AICc, second-order variant of Akaike’s Information Criterion; Δi, difference in AICc between model i and the top model; wi, relative likelihood of model i [i.e., Pr(modeli is the best model | data)]; k, number of model parameters. (PDF) [file pone.0149572.s003.pdf]

Rank[§]	Model	AIC_c	Δ_i	w_i	k
1 (1)	Colony presence + Water depth range + Alligator holes	890.26	0.00	0.55	5
2 (2)	Colony presence	891.86	1.59	0.25	3
3 (3)	Colony presence + Water depth range	894.35	4.09	0.07	4
4 (4)	Colony presence + Tree island area	894.45	4.19	0.07	4
5 (5)	Colony presence × Minimum water depth[†]	895.34	5.08	0.04	7
6 (7)	Tree island area	899.25	8.99	0.01	3
7 (6)	Water depth range	899.35	9.09	0.01	3
8 (9)	Minimum water depth[†]	900.76	10.49	0.00	4
9 (8)	Minimum water depth[†] × Water depth range	901.56	11.30	0.00	7
10 (10)	Minimum water depth[†] × Tree island area	904.37	14.11	0.00	7
11 (11)	Minimum water depth[†] × Alligator holes	906.69	16.43	0.00	7

§ Ranks in parentheses signify their position in model selection for Fulton's factor, *K*

† Quadratic term included
